# Intraoperative Cardiac Arrest during Zone 0 Thoracic Endovascular Aortic Repair in a Post-Coronary Artery Bypass Grafting Patient with Bilateral Internal Thoracic Artery Grafts

**DOI:** 10.3400/avd.cr.26-00067

**Published:** 2026-09-03

**Authors:** Keishi Miyazawa, Eriya Imai, Yasunori Iida, Tomomi Ueda, Tomoyuki Sato

**Affiliations:** 1 Department of Anesthesia, Saiseikai Yokohamashi Tobu Hospital, Yokohama, Kanagawa, Japan; 2 Division of Anesthesia, Mitsui Memorial Hospital, Tokyo, Japan; 3 Department of Social Medical Sciences, Graduate School of Medicine, International University of Health and Welfare, Tokyo, Japan; 4 Department of Cardiovascular Surgery, Saiseikai Yokohamashi Tobu Hospital, Yokohama, Kanagawa, Japan

**Keywords:** endovascular procedures, coronary artery bypass, ventricular fibrillation

## Abstract

An 85-year-old man, with a history of coronary artery bypass grafting (CABG) using bilateral internal thoracic artery (ITA) grafts, underwent zone 0 thoracic endovascular aortic repair (TEVAR) for an aortic arch aneurysm. The procedure involved axillary debranching and the combined use of conventional and fenestrated stent grafts. During deployment of the fenestrated graft, ventricular fibrillation occurred, temporally associated with a transient reduction in brachiocephalic artery blood flow that compromised ITA graft flow. He was discharged without neurological deficits. This case highlights the potentially critical risk of myocardial ischemia during zone 0 TEVAR in post-CABG patients, emphasizing the need for meticulous multidisciplinary planning.

## Introduction

Thoracic endovascular aortic repair (TEVAR) is an established treatment option for high-risk patients with aortic arch aneurysms due to its minimally invasive nature.^1)^ TEVAR can avoid a thoracotomy, cardiopulmonary bypass, and clamping of the aorta, resulting in lower postoperative mortality, fewer complications, and shorter hospital stays than open surgery.^2)^ In TEVAR, when covering arch branches, cervical debranching is required to maintain cerebral perfusion.^3)^ Additionally, a fenestrated stent graft is often deployed to secure the proximal landing zone to prevent endoleak. The anatomical landing zone between the ascending aorta and the brachiocephalic artery (BCA) is referred to as zone 0.^2)^

Aortic aneurysms and coronary artery disease often coexist, and aortic aneurysm repair may be required following coronary artery bypass grafting (CABG).^4)^ As CABG alters myocardial perfusion routes, the specific risk of myocardial ischemia caused by the deployment mechanism of fenestrated endografts during zone 0 TEVAR remains under-recognized. Although fenestrated stents are designed to maintain arch branch perfusion during their gradual expansion, transient and hemodynamically critical reductions in BCA flow can still occur, as illustrated by the present case. This rarely highlighted intraoperative interaction between the endograft deployment process and prior bypass grafts can lead to unexpected and potentially fatal events.

We report a case of ventricular fibrillation (VF) occurring during stent deployment in zone 0 TEVAR for an aortic arch aneurysm in a post-CABG patient.

## Case Report

**Fig. 1:**
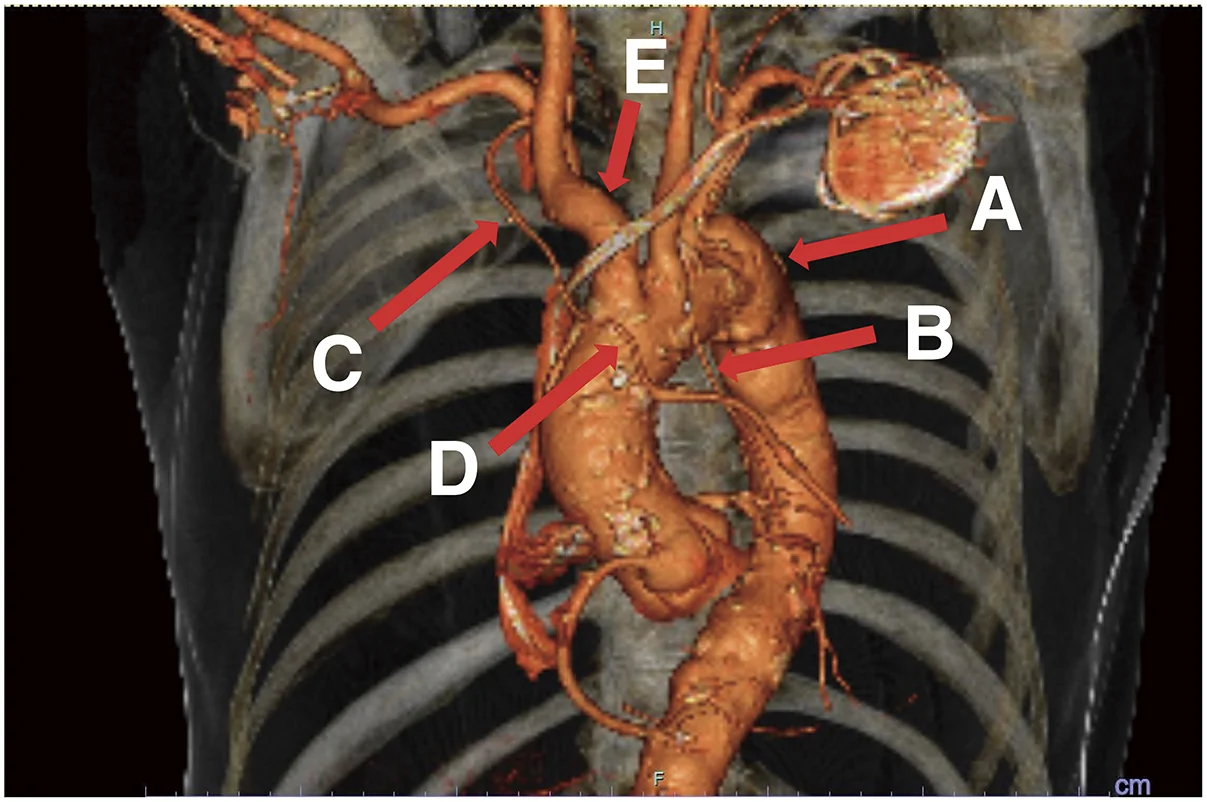
Preoperative reconstructive CT scan. (**A**) Aortic arch aneurysm. (**B**) Left internal thoracic artery (LITA). (**C**) Right internal thoracic artery (RITA). (**D**) Saphenous vein graft. (**E**) Brachiocephalic artery (BCA).

The patient was an 85-year-old man with an aortic arch aneurysm located immediately distal to the ostium of the left subclavian artery and involving the inner curvature of the arch (long diameter, 49 mm; maximum short diameter, 36 mm). He was independent in daily activities. The aneurysm had progressively enlarged over 10 yrs; surgical intervention was indicated due to the development of recurrent laryngeal nerve paralysis, which was impacting his daily communication. Asymptomatic arch aneurysms >55 mm are typically repaired; however, the indication here was progressive nerve paralysis from rapid aneurysmal expansion. He underwent 3-vessel CABG 10 yrs prior, with the left internal thoracic artery (LITA) to the left anterior descending artery, and a composite graft comprising the in situ right internal thoracic artery (RITA) and a saphenous vein graft to the high lateral branch. Computed tomography (CT) reconstruction is shown in **Fig. 1**. He had a permanent pacemaker for sick sinus syndrome (settings: ventricular-inhibited ventricular pacing with rate response; VVIR, heart rate; HR 70) and no history of VF or sustained ventricular tachycardia; this device had no defibrillation capabilities. He also had a history of atrial fibrillation. Transthoracic echocardiography revealed a left ventricular (LV) ejection fraction of 35% with diffuse LV hypokinesis and diastolic dysfunction.

**Fig. 2:**
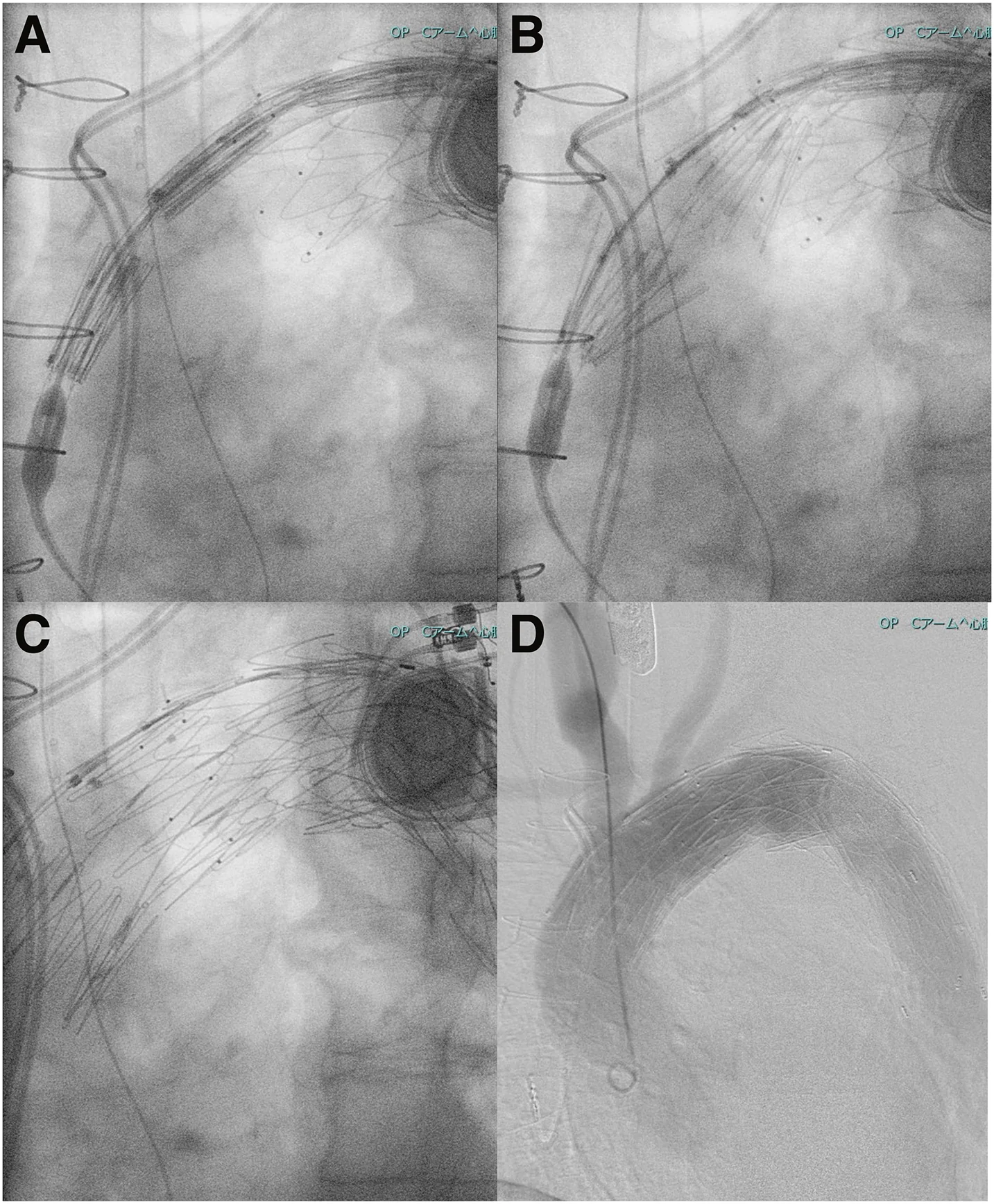
Intraoperative images of a Najuta stent. (**A**) Image showing a Najuta stent positioned in zone 0 after insertion. (**B**) Image showing the stent being gradually expanded. (**C**) Image after full deployment of a Najuta stent. (**D**) Post-deployment angiography.

TEVAR with axillary-to-axillary bypass was scheduled over open repair to avoid injury to the bypass graft vessel during re-do thoracotomy. The pacemaker showed total ventricular pacing and was set to ventricular asynchronous pacing mode (VOO) mode (HR 80) pre-induction. Arterial pressure was monitored via the left radial and right dorsalis pedis arteries. Total intravenous anesthesia was utilized with motor evoked potential (MEP), bispectral index (BIS), and regional cerebral oxygen saturation (rSO_2_) monitoring. After systemic heparinization, the axillary arteries were bypassed using an artificial graft (8-mm Gore Propaten Vascular Graft; W. L. Gore & Associates, Newark, DE, USA). Via percutaneous femoral artery access, a Relay Pro (36–36–150 mm; Terumo, Tokyo, Japan) was deployed in zone 2, followed by a Najuta Thoracic Stent Graft (40–36–175 mm, a fenestrated type; SB-Kawasumi Laboratories, Kanagawa, Japan) in zone 0 (**Fig. 2A**). Final angiography also confirmed patency of the coronary bypass grafts.

During the gradual deployment of the stent (**Fig. 2B**), blood pressure gradually declined to 62/40 (47) mmHg, unresponsive to phenylephrine, subsequently progressing to VF. Stent placement was expedited (**Fig. 2C**), and cardiopulmonary resuscitation (CPR) was initiated immediately. After 2 defibrillation attempts (150 J, 200 J) and 1 mg of intravenous adrenaline, return of spontaneous circulation (ROSC) was achieved within 4 min. Balloon molding was not performed during CPR. Transesophageal echocardiogram revealed no novel wall motion abnormality, valvular disease, or pericardial effusion. Arterial blood gas was unremarkable. Hemodynamic stability was achieved with no decline in MEP. During cardiac arrest, there was a profound drop in both BIS and rSO2 values, which returned to baseline after resuscitation. Once the patient’s condition stabilized, proper stent placement was verified (**Fig. 2D**), and a final angiography was subsequently performed to confirm the patency of the coronary bypass grafts.

The patient awoke promptly after surgery and was successfully extubated. Postoperative cerebral CT revealed no new lesions, and he was discharged on postoperative day 7 without neurological deficits. His hoarseness showed improvement early in the postoperative period.

## Discussion

This case reveals a critical risk of using a Najuta stent in zone 0 TEVAR in post-CABG patients: possible myocardial ischemia from a transient reduction of BCA and the coronary artery bypass graft flow during stent deployment.

Zone 0 TEVAR using a Najuta stent in post-CABG patients carries a risk of myocardial ischemia due to altered coronary perfusion, requiring careful management of hemodynamic changes during stent deployment. This patient’s coronary perfusion depended on LITA/RITA grafts, both of which received blood flow originating from the BCA after debranching. Bypassed internal thoracic artery (ITA) grafts are vulnerable to hypotension and vasospasm; maintaining flow over 20 mL/min is critical for adequate myocardial perfusion.^5)^ Once vasospasm occurs, these grafts show poor recovery and restriction of flow,^6)^ likely prolonging ROSC in this case. Although selective intraluminal administration of vasodilators is a potential strategy to relieve ITA vasospasm without inducing systemic hypotension,^6)^ achieving direct endovascular access to the RITA was technically difficult during this emergency scenario. Furthermore, systemic administration was not considered due to this patient’s profound hypotension and subsequent cardiac arrest. In typical Najuta stent placement, while trunk blood flow is interrupted, coronary flow from the aortic root is maintained. Hemodynamic compromise was temporally associated with the stent deployment mechanism, which transiently reduced cervical branch perfusion, as evidenced by decreasing left radial arterial pressure.

The combination of Relay Pro and Najuta stents in TEVAR for post-CABG patients can reduce aortic complications but risks myocardial ischemia from altered hemodynamics during zone 0 stenting. A standalone Relay Pro stent from zone 2 for a distal arch aortic aneurysm risks the bird-beak phenomenon and retrograde aortic dissection (RTAD).^7)^ Adding a Najuta stent proximally in zone 0 reduces these risks.^8)^ Najuta stents gradually open from the proximal end under native cardiac output, requiring approximately 2 min from initial expansion to full deployment.^9)^ Based on this sequential mechanism, we anticipated that any reduction in BCA flow through the fenestration would be minimal and well-tolerated. However, this case demonstrates that the gradual expansion process can cause a more profound decrease in perfusion than expected, acting as a temporary physical barrier. While such a temporary hemodynamic drop during deployment is usually well-tolerated in typical patients whose coronary perfusion originates from the aortic root, it proved catastrophic for this patient. Because his myocardial blood supply was entirely dependent on ITA grafts originating from the BCA, a transient reduction in BCA flow could be a possible mechanism for compromised coronary perfusion. Clinically, the gradual stent deployment was temporally associated with the onset of VF, raising the possibility that transient myocardial ischemia may have contributed to this catastrophic event. However, this proposed mechanism remains speculative because no direct objective evidence of myocardial ischemia was obtained. This uncertainty represents a limitation of our report. Nevertheless, the event suggests that such patients may have limited tolerance for even transient reductions in coronary perfusion. Preoperative evaluation can include an assessment of ITA graft dependency, typically via coronary angiography. Intraoperatively, the anesthesiologist can anticipate temporary arch branch flow compromise during stent deployment. Furthermore, continuous monitoring is paramount; this includes bilateral radial artery pressure monitoring to promptly detect isolated brachiocephalic or subclavian flow reductions, alongside cerebral rSO_2_ monitoring. Additionally, the proactive use of transesophageal echocardiography from the start of the procedure can be considered to continuously monitor for real-time signs of myocardial ischemia. To mitigate this life-threatening event, meticulous multidisciplinary planning involving cardiovascular surgery, anesthesiology, cardiology, nursing, and clinical engineering is indispensable.

In selected post-CABG patients with ITA grafts supplied via the BCA, preoperative planning should specifically assess whether the chosen deployment strategy could transiently compromise BCA flow. Furthermore, alternative strategies that preserve BCA perfusion should be considered when clinically feasible. For instance, a zone 1 TEVAR strategy with 2-stage debranching—right subclavian to left common carotid and left common carotid to left subclavian artery—may help maintain cardiac perfusion during stent deployment^10)^ while avoiding thoracotomy.

## Conclusion

This case highlights a critical risk of zone 0 TEVAR in post-CABG patients. Transient BCA occlusion during stent deployment likely compromises ITA graft perfusion and was temporally associated with the onset of VF. However, whether transient myocardial ischemia directly triggered VF remains speculative because no objective evidence of ischemia was obtained. This potentially life-threatening complication underscores the importance of meticulous preoperative planning and individual risk assessment to anticipate and mitigate these specific risks.
